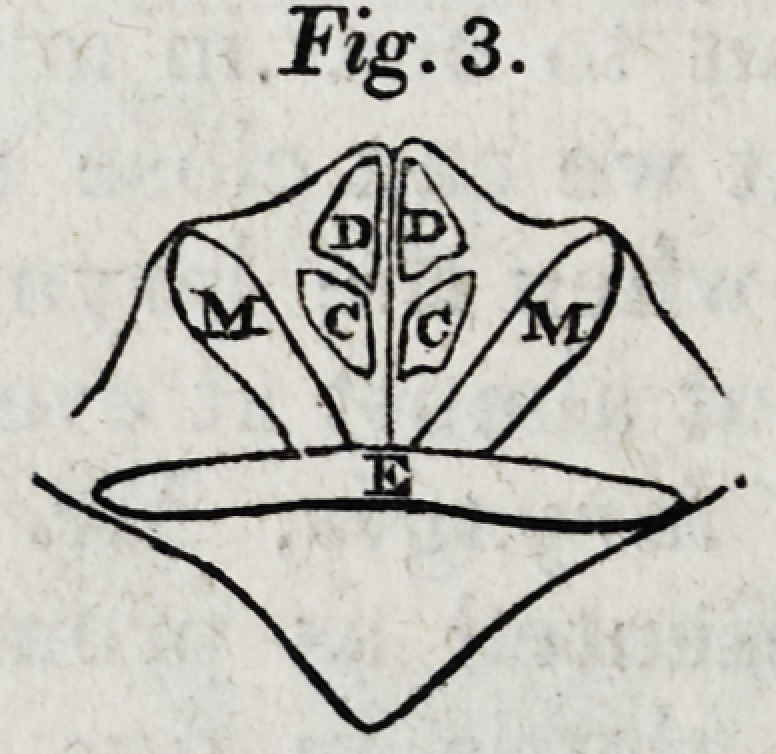# Description, by Mr. Mayo, of the Interior of the Larynx, as Seen after Attempted Suicide

**Published:** 1832-06

**Authors:** 


### INTERIOR OF THE LARYNX.

### *Description, by* Mr. Mayo, *of the Interior of the Larynx, as seen after attempted Suicide.*

Mr. Mayo has obliged us with a few more particulars respecting the case of a patient now under his care in the Middlesex Hospital, in whom the interior of the larynx is partially visible through a wound made in attempted suicide. These we publish with the rest of the case, as it appeared in the Medical Gazette.

T. P., ætat. fifty, was admitted into the Middlesex Hospital, on the 4th of April: he had attempted to destroy himself by inflicting a deep wound on the fore part of the throat, between the os hyoïdes and the thyreoïd cartilage. The epiglottis had been divided immediately above the thyreoïd, so that the upper part of the arytænoïd cartilages was exposed. The breadth of the opening into the pharynx exceeded an inch; no large blood-vessel had been wounded.

The treatment of this patient, who is rapidly recovering, has been of the simplest nature. Any attempt to keep the surfaces of the wound in contact has been found to give great distress, by preventing the free escape of the mucus which accumulates in the throat, and which the patient is unable to get rid of through the fauces. The wound, left to itself, with the exception of continual cleansing, is covered with healthy granulations, and contracts daily.

The following are the physiological observations made by Mr. Mayo on this case.

1. Deglutition is performed without difficulty: only, when liquids are swallowed, a small quantity escapes through the wound. This is not attended with coughing, or any sensible irritation of the larynx.

2. All that can be seen of the interior of the larynx are the upper part of the arytænoïd cartilages, and the folds of membrane reflected from thence towards the epiglottis. During gentle respiration, the state of these parts is exactly such as is represented in Fig. 1; in which S is the granulating inferior surface of the wound; P, the posterior surface of the pharynx; and T, the membrane covering the fore part of the arytænoïd cartilages.

*Fig.* 1.

The aperture of the glottis, under these circumstances, is seen to be considerably expanded and motionless. It does not, however, continue motionless if the patient become agitated, and the breathing disturbed: in the latter case, at each expiration, the aperture is considerably narrowed.

3. When the patient forcibly, and with an effort of straining, closes the glottis, the appearance of the parts is such as is represented in Fig. 2.

*Fig.* 2.

The median vertical line is now very distinctly observable, at which the arytænoïd cartilages are pressed against each other. The superior aperture of the glottis, though narrowed, is open. The closure of the glottis takes place not at its upper border, but at the superior ligaments.

4. When the patient utters a continued sound, the condition of the parts appears to be exactly the same as when the glottis is forcibly closed; or rather, when the patient passes alternately from one of these actions to the other, now closing the glottis, now uttering a continued sound, no sensible change takes place in the disposition of the parts. The arytænoïd cartilages do not appear more or less closely applied to each other in either case; only, while sound is uttered, the whole surface appears tremulous with vibration.

5. The patient whose case we are considering, (contrary to Mr. Mayo's first observation,) can now utter notes of different pitch. When he utters a higher note, the following changes are observed: the larynx rises, and the interval between the *cornua laryngis* is greater than when the note is lower; as if the upper parts of the arytænoïd processes were on this occasion not so closely applied to each other as before. It is important to bear in mind, in connexion with these observations, that we can close the glottis by a voluntary effort, not merely when the larynx is at its highest elevation in the throat, but almost at every other.

By the term *cornua laryngis*, Mr. Mayo means the two bodies which are represented as prominent at the posterior and upper part of the glottis in figs. 1 and 2; of these he gives the following account.

"In each of these figures two strongly projecting parts are drawn, the distance or approximation of which marks the open or closed state of the glottis. I was at first at a loss to understand what these prominent parts were; but, upon carefully examining the larynx in an adult male body, and comparing it with the appearances in the present case, I found that they must be the cuneïform cartilages.

"These parts have not, that I know of, been described in their true shape and magnitude: I have therefore in Fig. 4 represented the cuneïform cartilage of one side with the

*Fig. 4.*

other cartilages and the ligaments of the glottis, as they are seen from within on a vertical section of the larynx.

"The cuneïform cartilages in the specimen from which these drawings were made, were (as is most usual) upwards of half an inch in length; their form nearly cylindrical, and their diameter four-fiftieths of an inch. At their inferior ends they became thinner, and were connected with the elastic fibres of the ligamenta superiora glottidis, where the latter are attached to the arytænoïds. In the larynx of a girl of fourteen, their inferior ends were larger even than the upper. The term *cornua laryngis* would be more appropriate to them than that of cuneïform cartilages."

*Fig.* 3.

In Fig. 3, the reader will find a front view of the upper and back part of a dissected larynx, shewn much in the same manner (through the division of the thyreoïd,) as in the wound in the living patient; by which he may easily identify the appearances in the Figs. 1 and 2 with the parts as represented in Fig. 4.

The letters in Fig. 3 and 4 designate—

A. The thyreoïd cartilage.
B. The cricoïd.
C. The arytænoïd.
D. The corniculum laryngis.
E. The epiglottis.
I. The ligamentum inferius glottidis.
K. The ligamentum superius.
M. The *cornu laryngis*.

## Figures and Tables

**Fig. 1. f1:**
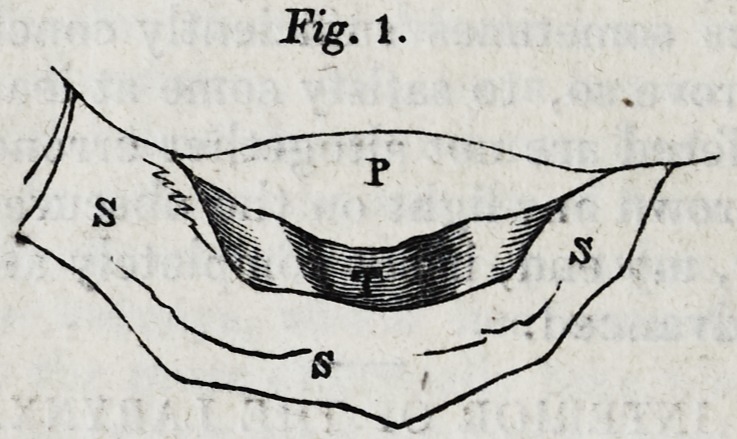


**Fig. 2. f2:**
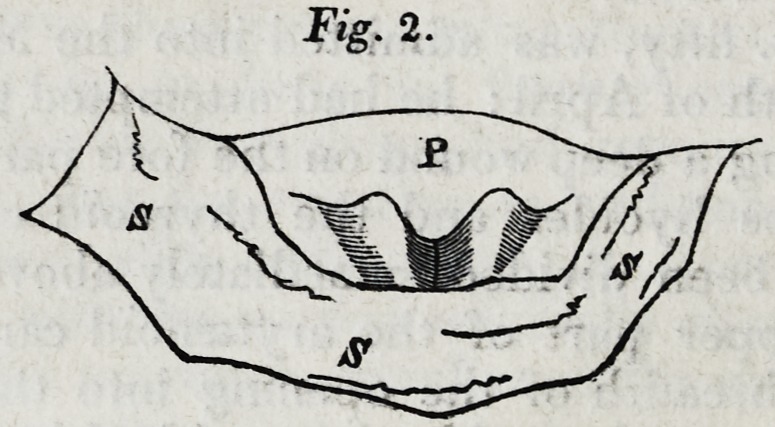


**Fig. 4. f3:**
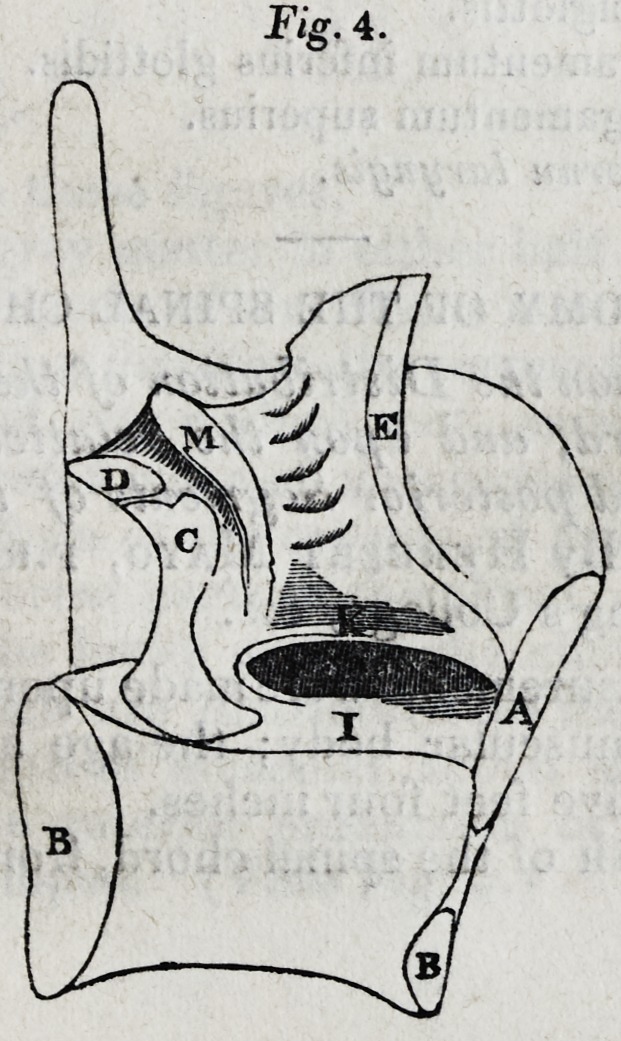


**Fig. 3. f4:**